# Cost Effectiveness of Seasonal Intermittent Preventive Treatment Using Amodiaquine & Artesunate or Sulphadoxine-Pyrimethamine in Ghanaian Children

**DOI:** 10.1371/journal.pone.0012223

**Published:** 2010-08-17

**Authors:** Lesong Conteh, Edith Patouillard, Margaret Kweku, Rosa Legood, Brian Greenwood, Daniel Chandramohan

**Affiliations:** 1 London School of Hygiene and Tropical Medicine, London, United Kingdom; 2 Imperial College London, London, United Kingdom; 3 Ghana Health Service, University of Ghana, Legon, Accra, Ghana; Erasmus University Rotterdam, Netherlands

## Abstract

**Background:**

Intermittent preventive treatment for malaria in children (IPTc) involves the administration of a full course of an anti-malarial treatment to children under 5 years old at specified time points regardless of whether or not they are known to be infected, in areas where malaria transmission is seasonal. It is important to determine the costs associated with IPTc delivery via community based volunteers and also the potential savings to health care providers and caretakers due to malaria episodes averted as a consequence of IPTc.

**Methods:**

Two thousand four hundred and fifty-one children aged 3–59 months were randomly allocated to four groups to receive: three days of artesunate plus amodiaquine (AS+AQ) monthly, three days of AS+AQ bimonthly, one dose of sulphadoxine-pyrimethamine (SP) bi-monthly or placebo. This paper focuses on incremental cost effectiveness ratios (ICERs) of the three IPTc drug regimens as delivered by community based volunteers (CBV) in Hohoe, Ghana compared to current practice, i.e. case management in the absence of IPTc. Financial and economic costs from the publicly funded health system perspective are presented. Treatment costs borne by patients and their caretakers are also estimated to present societal costs. The costs and effects of IPTc during the intervention period were considered with and without a one year follow up. Probabilistic sensitivity analysis was undertaken to account for uncertainty.

**Results:**

Economic costs per child receiving at least the first dose of each course of IPTc show SP bimonthly, at US$8.19, is the cheapest to deliver, followed by AS+AQ bimonthly at US$10.67 and then by AS+AQ monthly at US$14.79. Training, drug delivery and supervision accounted for approximately 20–30% each of total unit costs. During the intervention period AS & AQ monthly was the most cost effective IPTc drug regimen at US$67.77 (61.71–74.75, CI 95%) per malaria case averted based on intervention costs only, US$64.93 (58.92–71.92, CI 95%) per malaria case averted once the provider cost savings are included and US$61.00 (54.98, 67.99, CI 95%) when direct household cost savings are also taken into account. SP bimonthly was US$105.35 (75.01–157.31, CI 95%) and AS & AQ bimonthly US$211.80 (127.05–399.14, CI 95%) per malaria case averted based on intervention costs only. The incidence of malaria in the post intervention period was higher in children who were <1 year old when they received AS+AQ monthly compared to the placebo group leading to higher cost effectiveness ratios when one year follow up is included. The cost per child enrolled fell considerably when modelled to district level as compared to those encountered under trial conditions.

**Conclusions:**

We demonstrate how cost-effective IPTc is using three different drug regimens and the possibilities for reducing costs further if the intervention was to be scaled up to the district level. The need for effective training, drug delivery channels and supervision to support a strong network of community based volunteers is emphasised.

## Introduction

It is estimated that 300–500 million malaria episodes are recorded each year, 90% of which occur in sub Saharan Africa, causing approximately 800,000 deaths [1], [2]. In addition to its impact on the health of individuals, malaria places considerable costs on households [3]–[5], communities[6] and nations [7], [8]. In Ghana, the setting of this study, malaria is the leading cause of morbidity, accounting for 40–60% of outpatient visits and it is the leading cause of mortality in children under five[9].

Intermittent preventive treatment for malaria in children (IPTc) is a promising new approach to malaria control in areas where malaria transmission is seasonal[10]. IPTc involves the administration of a full course of an anti-malarial treatment to children at specified time points regardless of whether or not they are known to be infected. Studies of IPTc conducted in Senegal and Mali have shown that IPTc with sulphadoxine-pyrimethamine (SP) plus artesunate (AS), SP plus three daily doses of amodiaquine (AQ) or SP alone is an efficacious and safe intervention for reducing the burden of malaria in children in high transmission areas with short transmission periods[10]–[12]. A recent study in Senegal suggests that seasonal IPTc with SP plus piperaquine is highly effective and well tolerated[13].To date IPTc has been shown to be efficacious in reducing the incidence of malaria in areas with a short malaria transmission season. The purpose of this study in Hohoe, Ghana was to investigate the ability of IPTc to reduce the burden of malaria in an area with a more prolonged transmission season.

In addition to showing the efficacy and effectiveness of IPTc, it is important to determine the costs associated with IPTc in terms of the total expenditure needed to provide IPTc and also the potential savings to providers and caretakers achieved by reducing the number of children who present at health facilities for inpatient or outpatient care.

The findings in this paper are based on a randomised, placebo controlled trial designed to evaluate the effectiveness of IPTc in reducing anaemia and malaria in an area with up to 6 months of transmission in Ghana. Two thousand four hundred and fifty-one children aged 3–59 months were randomly allocated to four groups to receive: three treatments with artesunate plus amodiaquine (AS+AQ) monthly, three with AS+AQ bimonthly, one dose of SP given bi-monthly or placebo. Compared to placebo, trial results showed that monthly AS+AQ reduced the incidence of malaria by 69% (95% CI: 63%, 74%) and anaemia by 45% (95% CI: 25%,60%), bimonthly AS+AQ reduced the incidence of malaria by 17% (95% CI: 6%, 27%) and anaemia by 32% (95% CI: 7%, 50%) and bimonthly SP reduced the incidence of malaria by 24% (95% CI: 14%,33%) and anaemia by 30% (95% CI: 6%, 49%)[14]. This paper focuses on incremental cost effectiveness ratios (ICERs) of the three IPTc drug regimens as delivered by community based volunteers (CBV) in Hohoe, Ghana compared to current practice, i.e. case management in the absence of IPTc. It is the first cost effectiveness analysis of IPTc to our knowledge. The cost analysis is from the perspective of the publicly funded health system and includes both financial and economic costs. Treatment costs borne by patients and their caretakers are also estimated to present societal costs.

## Methods

### Ethics Statement

The study was approved by ethical committees of the Ghana Health Service/Ministry of Health (GHS/MOH) and the London School of Hygiene & Tropical Medicine. After obtaining written, informed consent, data on malaria treatment costs were collected from the families of children involved in the study. Verbal consent was sought from the health care professionals before they were asked, or observed, in an attempt to assess the resource use and associated costs of treating children with malaria. Verbal consent was considered sufficient for health care workers as the socio economic analysis came at the end of the wider efficacy study and thus facility staff had a history of working with those in the study and were already sensitized to the aims and objectives of the cost effectiveness sub study. The ethics committees in Ghana and London were made aware of the use of both verbal and written consent.

### Study Area and Population

This study was carried out in Hohoe district, Ghana. A full description of the study area has been published elsewhere [14]. The transmission of malaria in the area is intense with two seasonal peaks. The major wet season lasts from April to July and the minor one from September to November. The entomological inoculation rate during the study period was approximately 65 infective bites per person per year (unpublished data). The fist line treatment drug in Ghana has been amodiaquine plus artesunate since 2006.

### Calculating Effectiveness

The study was a randomized, placebo-controlled trial of IPTc conducted in children aged 3–59 months who resided in the study district. The trial took place during the six months of the high malaria transmission season. From April to September 2005, 2451 children were randomised to receive one of the following three IPTc regimens under direct supervision of the CBVs every 28 days (placebo or active drug): (i) a single dose of SP every two months, (ii) a three-day course of AS+AQ every two months and (iii) a three-day course of AS+AQ every month. All single and multiple doses of SP and AS+AQ respectively were administered by the CBVs. Children were followed for one year after stopping IPTc to monitor the possibility of a rebound in malaria morbidity.

Field workers visited study children once a week during the period of drug administration to enquire about their health and completed a morbidity form if a child had any illness. A passive surveillance system to monitor malaria and anaemia in study children throughout the study period was set up in the district hospital and in 21 health centres in the study area. If a child who presented at one of these facilities had fever or any features suggestive of malaria, a finger prick blood sample was collected for malaria parasite examination before treatment was given. Blood slides were read at the respective health facility to decide on treatment and read again in a central laboratory to confirm the diagnosis. Children with proven or presumptive malaria were treated with oral quinine according to the Ministry of Health (MOH) treatment guidelines.

### Calculating Costs

Three main areas of costing were determined - the cost of delivering IPTc, the cost of malaria case management from the provider perspective (inpatient and outpatient visits to a government facility) and the costs incurred by the caretakers of the children visiting the inpatient and outpatient facilities. Care was taken to exclude resources related to research activities. Financial and economic total costs are presented. Financial costs reflect the additional resources required to deliver IPTc in terms of the actual expenditures incurred. For example the payment of resources such as IPTc drugs, incentives, materials and supplies. Straight line depreciation of capital costs is used in the financial cost estimates. The economic costs capture the opportunity cost of all resources used to provide IPTc, whether or not they incur a financial cost. For example, the time health personnel are involved in IPTc delivery represents an economic cost to the programme, because although they are already receiving a salary from the Ministry of Health, they could have spent their time in other activities. An annualisation and discount rate of 3% is used to calculate economic costs[15].

#### Costs of IPTc

The cost of delivering (i.e. ensuring the supply of IPTc drugs from central medical stores to the CBV) and administering IPTc during the six months of the intervention was identified using components of the trial budget and data collected on resource use. Costs categories included those of IPTc drugs, training of health personnel and CBVs, health personnel staff time, utilities (such as any water, gas, electricity and telephone bills), supplies, transport supervision and incentives. Children aged 3–5 months received a quarter of a tablet, those aged 6–11 months half a tablet, those aged 12–23 months three quarters of a tablet and those aged 24 months and above received one tablet each of SP, co-formulated AS+AQ or placebo. The full tablet was costed across all age groups to allow for wastage.

The volunteers who had been selected by the caretakers of the children in each community to administer the IPTc drugs were paid an allowance of approximately US$10 a month. This was based on the amount paid to volunteers who administer vaccines during poliomyelitis, measles and neonatal tetanus campaigns (US$3 per day for three days).The training of volunteers took place at the Hohoe District Health Directorate for five days. The volunteers were trained on how to identify drugs packaged for each child, administer drugs, complete drug administration and morbidity forms and when to refer patients to the health facility. Two field supervisors visited the volunteers to provide support and supervise the administration of every first dose of drug administration. They also visited the each volunteer every week to collect adverse event and morbidity forms. They used the forms to follow-up the adverse event cases.

Health personnel costs associated with IPTc delivery were obtained from the 2005 records of salaries and allowances and 2006 consolidated salaries paid at the Hohoe district hospital and district health centres. Although not collected in Hohoe, the cost to the caretaker of accessing IPTc was explored in a similar study in The Gambia and found to be negligible (unpublished data).

Having undertaken a detailed costing of delivering IPTc to the study population, the costs of scaling up the intervention to Hohoe district were modelled. This involved increasing the potential number of children receiving IPTc from 2451 (total number in the trial) to 33,000 (total number of under fives in the district). Resources used and their associated costs were based on information from the study budget, other community based programmes such as poliomyelitis, measles and neonatal tetanus campaigns underway in the Hohoe district and estimates form the principal investigator involved in the original IPTc clinical trial.

#### Provider costs of malaria treatment

Provider costs were based on detailed retrospective cost data obtained from government facilities. Costs were identified using records supplied by the Ministry of Health and the Ministry of Finance, together with components of the study budget and patient folders in the study site in Hohoe. An ingredients approach was used and costs included personnel, materials and supplies, transport, utilities and buildings [16], [17].

Staff time required for the management of children with clinical malaria and/or anaemia was estimated by direct observation and interviews undertaken with staff at the hospital and health centres. Once verbal consent was obtained from the health workers and parent/guardian of patient, children were tracked from the time of arrival at the out-patients department and time spent with each health worker was recorded. More general costs were apportioned based on the number of under 5 year old malaria (severe or clinical) cases as a proportion of the total number of outpatient and inpatient visits recorded at the facilities.

#### Household costs of malaria treatment

Having given informed consent, structured interviews with primary caretakers of children were conducted to determine any costs related to malaria treatment incurred at the household level. All children in the IPTc study who visited the outpatient department during the intervention period with clinical malaria or malaria with anaemia were visited at home up to 12 months after the intervention. Only 207 out of the 448 (46%) children were available at the time of the survey. Ten out of 44 (25%) of those admitted to the hospital with a diagnosis of malaria or malaria with severe anaemia were also interviewed at home. Families of patients incurred both indirect and direct costs. Direct costs included out-of-pocket expenses on items such as formal hospital fees (admission fees, blood transfusions), informal hospital fees (locally known as the welfare ward fund commonly associated with outpatient visits), medication, food and transport costs. Due to the long recall period, once the mode of transport used by the career and the sick infant was identified a standard fare was applied to the number of journeys reported (based on reported Ghana private road and transport union standard taxi and bus fares). Similarly the market rate for an adult meal was used as a proxy for the direct cost of food. Drug costs were calculated using the records of study patients who had received inpatient or outpatient care respectively. The drug costs borne by the household were cross referenced with costs identified at the district hospital pharmacy, the district health directorate store and published market drug prices [18]. Indirect costs included time lost as a result of caring for a sick child at home, traveling to hospital, and the time spent at the facility while the child in their care was receiving treatment. Time lost was valued at the prevailing minimum subsistence wage rate in Ghana in 2005 (i.e. 15,200 old Ghanaian Cedis/1.68 US$ per day). This is one approach to costing lost productivity amid a lack of consensus on how best to value unpaid work[19].

Care was taken to avoid double counting costs when combining both provider and household direct costs as certain costs were considered cross subsidisations, for example the admission fees paid by the household was a transfer of funds to subsidise certain provider costs. Using the total costs (Table 1) and later the unit costs (Table 2) of the intervention, the unit costs associated with treating malaria (Table 3), and the effectiveness data (Table 4), the estimates of cost effectiveness of IPTc during the intervention time and up to one year after its completion are presented (Table 5).

**Table 1:** Total Costs of Delivering different IPTc regimens(US$ 2008).^a^

	SP Bimonthly (n = 613 children)				AQ & AS Monthly (n = 626 children)				AQ & AS Bimonthly (n = 562 children)			
	Financial		Economic		Financial		Economic		Financial		Economic	
	Total Costs	Cost Profile	Total Costs	Cost Profile	Total Costs	Cost Profile	Total Costs	Cost Profile	Total Costs	Cost Profile	Total Costs	Cost Profile
Cost of IPTc Drugs	73	3%	73	2%	1170	18%	1170	14%	525	15%	525	10%
Drug Administration (CBVs)	136	5%	136	3%	814	12%	814	10%	407	11%	407	8%
Drug Delivery^b^												
* Personnel*	511	20%	511	12%	1022	16%	1022	12%	511	14%	511	10%
* Transport*	254	10%	254	6%	509	8%	509	6%	254	7%	254	5%
Supervision												
* Personnel*	531	21%	531	12%	1743	27%	1743	21%	871	24%	871	16%
* Transport*	231	9%	231	5%	572	9%	572	7%	286	8%	286	5%
Training												
* CBVs*	444	17%	488	11%	374	6%	418	5%	408	11%	452	9%
*DHMT & surveillance staff*	320	12%	1967	46%	270	4%	1916	23%	294	8%	1940	37%
Supplies	71	3%	71	2%	72	1%	72	1%	65	2%	65	1%
**Total Cost:**	**2572**	**100%**	**4262**	**100%**	**6546**	**100%**	**8237**	**100%**	**3622**	**100%**	**5312**	**100%**

a There was an imbalance in the number of children allocated to one study group. All possible reasons as to why this might have occurred were explored and chance was the most likely explanation [14].

b Drug delivery refers to the supply channel from the central medical stores to the community based volunteer.

**Table 2:** Unit Costs of receiving IPTc (cost per child receiving at least the first dose of each course*) (US$2008).

	SP Bimonthly		AQ & AS Monthly		AQ & AS Bimonthly	
	Economic Unit Cost	Cost Profile	Economic Unit Cost	Cost Profile	Economic Unit Cost	Cost Profile
Cost of IPTc Drugs	0.13	2%	2.04	14%	1.02	10%
Drug Administration (CBVs)	0.26	3%	1.47	10%	0.82	8%
Drug Delivery						
* Personnel*	0.98	12%	1.84	12%	1.03	10%
* Transport*	0.49	6%	0.92	6%	0.51	5%
Supervision						
* Personnel*	1.02	12%	3.15	21%	1.76	16%
* Transport*	0.44	5%	1.03	7%	0.58	5%
Training**						
* CBVs*	0.94	11%	0.75	5%	0.91	9%
* DHMT & surveillance staff*	3.78	46%	3.46	23%	3.91	37%
Supplies	0.14	2%	0.13	1%	0.13	1%
**Total Cost:**	**8.19**	**100%**	**14.79**	**100%**	**10.67**	**100%**

*A dose reflects the act of the child taking each daily IPTc tablet (one day for SP and three days for AQ& AS), a course reflects the monthly taking of all doses of IPTc.

**The slight differences in training costs reflect the different number of children assigned to each trial arm.

**Table 3:** Unit Costs of Inpatient and Outpatient Treatment of Malaria with and without Anaemia (US$ 2008).

	Outpatient Costs				Inpatient Costs			
	Clinical Malaria		Clinical Malaria & Anaemia		Severe Malaria		*Severe Malaria & Severe Anaemia*	
***PROVIDER COSTS***								
Personnel costs	2.66	32%	2.73	25%	18.57	24%	32.67	*17%*
Material & Supplies costs	0.01	0%	0.01	0%	7.79	10%	30.30	*16%*
Transport cost:	0.02	0%	0.02	0%	0.02	0%	0.02	*0%*
Utilities cost:	0.16	2%	0.16	2%	0.83	1%	0.83	*0%*
Building costs	0.03	0%	0.03	0%	0.26	0%	0.26	*0%*
*Subtotal Provider Costs:*	*2.88*	*34%*	*2.94*	*27%*	*27.48*	*35%*	*64.08*	*34%*
								
***HOUSEHOLD COSTS***								
Fixed Consultation Fee	0.60	7%	0.60	6%	0.60	1%	0.60	*0%*
Antimalarial drug costs:	0.94	11%	1.80	17%	0.23	0%	20.69	*11%*
Non-malarial drug costs:	0.74	9%	0.74	7%	0.07	0%	1.57	*1%*
Additional Medical Expenses	0.00	0%	0.00	0%	11.67	15%	45.44	*24%*
Food	1.81	21%	1.81	17%	11.14	14%	11.14	*6%*
Transport cost:	0.34	4%	0.34	3%	0.45	1%	1.06	*1%*
Welfare Fee	0.12	1%	0.12	1%	-	0%	-	*0%*
*Subtotal Direct Household Costs*	*4.56*	*54%*	*6.91*	*64%*	*31.30*	*40%*	*80.50*	*43%*
*Subtotal Indirect Household Costs* *	*1.00*	*12%*	*1.00*	*9%*	*19.15*	*25%*	*42.30*	*23%*
*Subtotal All Household Costs*	*5.55*	*66%*	*7.91*	*73%*	*50.46*	*65%*	*122.79*	*66%*
**TOTAL UNIT COSTS**								
**Provider Costs**	**2.88**		**2.94**		**27.48**		**64.08**	
**Provider and Direct Household Costs** **	**6.82**		**7.76**		**39.36**		**98.49**	
***Provider and Direct and Indirect Household***	**7.83**		**8.75**		**58.51**		**140.83**	

*Lost productivity.

**Care has been taken to avoid double counting certain provider and household costs.

**Table 4:** Effectiveness of different IPTc regimens.

	Totals				Cases Averted		
	Placebo	SP Bimonthly	AS & AQ Monthly	AS & AQ Bimonthly	SP Bimonthly	AS & AQ Monthly	AS & AQ Bimonthly
**(a) During Six Month Intervention Period**							
No. of children enrolled in start of 6 months	650	613	626	562	-	-	-
No. of children involved at end of 6 months	613	562	594	522	-	-	-
Clinical malaria only	119	79	18	67	40	101	52
Clinical malaria & anaemia	64	33	26	42	31	38	22
Severe malaria only	12	10	3	6	2	9	6
Severe malaria & severe anaemia	7	1	4	1	6	3	6
	Protective Efficacy 95% CI						
Malaria with any parasitaemia		24.3 (14.1−33.4)	69.1 (62.9−74.2)	17.4 (6.3−27.2)			
All cause admissions		−16.7 (−142.−39.9)	0.0 (−222−53.7)	−12.5 (−194–66.9)			
Malaria admissions		7.4 (−199.−71.4)	15.1 (−281.8−81.1)	−10.5 (−815−55.9)			
**(b) Intervention period and One Year Follow Up (includes rainy and dry season)**							
No. of children followed up	589	550	559	464	-	-	-
Clinical malaria only	162	133	75	103	29	87	59
Clinical malaria & anaemia	79	54	42	53	25	37	26
Severe malaria only	241	187	117	156	54	124	85
Severe malaria & Severe anaemia	15	12	10	10	3	5	5

**Table 5:** Cost Effectiveness of IPTc.

	During Six Month Intervention Period			*Intervention Period & One Year Follow Up*		
	SP Bimonthly	AS & AQ Monthly	AS & AQ Bimonthly	SP Bimonthly	AS & AQ Monthly	*AS & AQ* Bimonthly
***Gross Intervention Costs***						
***Based on Intervention Costs only***						
Cost per child enrolled	6.95	13.16	9.45	-	-	*-*
Cost per child fully adherent	8.19	14.79	10.67	-	-	*-*
Cost per malaria case averted - clinical cases only	105.35 (75.01−157.31	67.77 (61.71−74.75)	211.80 (127.05−399.14)	-	-	*-*
Cost per malaria case averted - clinical & severe cases	107.46 (74.83 −157.92)	72.18 (59.37–85.05)	207.51 (121.21−395.27)	-	-	*-*
***Resources Savings*** *						
Provider	(206)	(402)	(214)	(115)	(356)	(244)
Provider and Direct Household	(513)	(984)	(526)	(392)	(881)	(604)
Provider and Direct and Indirect Household	(584)	(1123)	(600)	(446)	(1005)	(689)
**Net Costs** **						
Provider	4056	7835	5098	4017	7881	5068
Provider and Direct Household	3749	7253	4787	3871	7356	4708
Provider and Direct and Indirect Household	3678	7114	4713	3817	7232	4623
**Net Cost Effectiveness (Cost per malaria case averted)** ***						
Provider	102.33 (72.71–153.00)	64.93 (58.92–71.92)	208.69 (125.5–391.20)	-	-	*-*
Provider and Direct Household	98.39 (68.77–149.04)	61.00 (54.98–67.99)	204.75 (121.60–387.26)	-	-	*-*

Numbers in parenthesis are cost savings.

*This is based on the savings of averting treatment compared to the placebo (See Table 3).

**Net costs are calculated by subtracting resource savings from intervention costs.

***Net costs effectiveness is calculated by dividing costs by the protective efficacy against clinical malaria.

Costs and incremental cost effectiveness ratios (ICERs) are expressed in US$ 2008. Expenses were converted into the local Ghanaian Cedis. An average value of the Cedi was taken over the time of the analysis. This translated into costs spanning from March 2005 until November 2005, with an average of Cedis 9074 =  1US$ (ranging from Cedis 8617 to Cedis 9150) [18]. The costs based in 2005 were then inflated to 2008 using US inflation rates for tradable goods and Ghanaian inflation rates for non tradable goods [20].

ICERs were calculated as probability distributions rather than as point estimates; 10,000 iterations were run[21]. Normal distribution was used for the original trial efficacy data and rates of events combined with the observed cost estimates. In addition, cost effective acceptability curves (CEACs) are presented for each of the drug regimens to help demonstrate the likelihood that IPTc would be cost effective at various levels of willingness to pay compared to current practice without IPTc for a range of values of λ[22]. A cost effectiveness plane is presented to explore if one of the IPTc drug regimens is dominant.

## Results

The total costs of giving SP bimonthly to 613 children, AS+AQ monthly to 626 children and AS+AQ bi-monthly to 562 study children are presented in Table 1. As would be expected, SP bimonthly costs the least to deliver, followed by AS+AQ bi-monthly and then AS+AQ monthly. The most significant difference in the economic and financial costs appears in the training cost centres. The financial costs of training refer to the per diems and expenditure spent directly on facilitating the training. Economic costs also include the opportunity cost (the salaries) of everyone involved in the training, accounting for the time that they were unable to carry out their usual duties.

Table 2 divides the total costs by the number of children who received at least the first dose of each monthly course of IPTc by drug regimen. In Table 2, and subsequent results, only the economic costs are presented as these represent the true costs of delivering IPTc. SP bimonthly, at US$8.19, is the cheapest to deliver, followed by AS+AQ bimonthly at US$10.67 and then by AS+AQ monthly at US$14.79. As to be expected, doubling the frequency of delivering the intervention did not double the unit costs, as activities such as training were a fixed cost regardless of the frequency of the intervention. The cost of the AS+AQ drug was considerably higher than SP. Training, drug delivery and supervision accounted for approximately 20–30% each of total unit costs.

The unit costs of an inpatient or outpatient visit for malaria with or without anaemia are presented in Table 3. The majority of costs of treating malaria are borne by the household and not by the health provider. The average cost to the provider of treating a case of uncomplicated clinical malaria or malaria with anaemia as an outpatient was US$2.88 and US$2.94 respectively and the costs of treating severe malaria or severe malaria anaemia were US$27.48 and US$64.08 respectively. These costs increased substantially when direct out of pocket household costs are added due to the costs of drugs and medical supplies such as purchasing blood to treat severe anaemia. Outpatient costs for both the health care provider and household directly were US$6.82 and US$7.76 for treating uncomplicated clinical malaria and malaria with anaemia respectively whilst the costs of inpatient care was US$39.36 for severe malaria alone and US$98.49 for malaria with severe anaemia. Treatment cost increased even further when indirect costs associated with lost productivity were added.

Table 4 presents the effectiveness results from the trial [14]. Note that there were no statistically significant reductions in episodes of all cause or malaria specific hospital admissions in any of the intervention groups. ICERs based on the statistically significant impact IPTc had on clinical (outpatient) malaria only, had narrower confidence intervals, but were not very different from the ICERs that included both clinical and severe (inpatient) episodes, due to the small number of severe cases averted. The estimates of cost effectiveness of IPTc during the intervention time and during the year after its completion are presented in Table 5. There are three main areas worth noting in this table. Firstly, although AS & AQ monthly is the most costly IPTc regimen to deliver (at US$13.16 per child enrolled or US$14.79 per child fully adherent, it is the most cost effective option given its substantially higher protective efficacy. Secondly, the cost effectiveness ratios decrease when the costs incurred not only by providers but also by household are included. Thirdly, there was no significant increase in the incidence of clinical malaria in the post intervention period in children who were >1 year old when they received IPTc compared to the placebo group. However the incidence of malaria in the post intervention period was higher in children who were <1 year old when they received AS+AQ monthly compared to the placebo group [14]. This is shown by the slight increase in treatment costs and increase in the net costs of IPTc for SP bimonthly and AS+AQ bimonthly when findings are compared for the intervention period to alone with those that also include the one year follow up.

**Figure 1:**
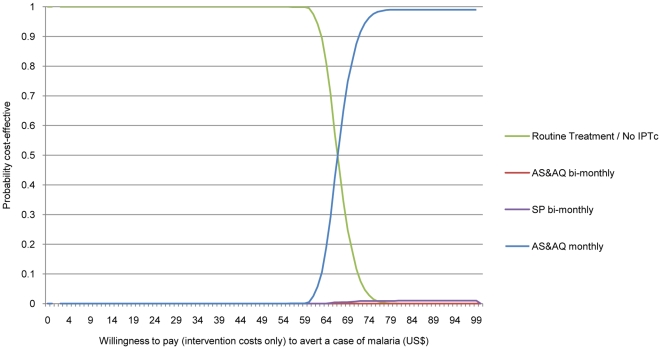
Cost-Effectiveness Acceptability Curve: Probability that IPTc is cost-effective given willingness to pay to avert a case of malaria.

**Figure 2:**
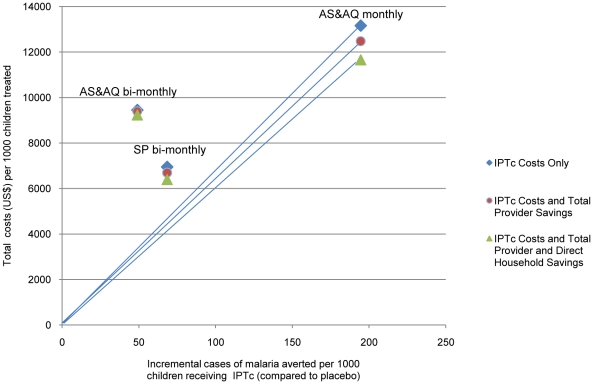
Cost-Effectiveness Plane: Incremental costs and cases of malaria averted per 1000 children receiving IPTc compared to no IPTc.

The acceptability curves of the three IPTc drug regimens are presented in Figure 1 depicting the cumulative distribution of cost-effectiveness ratios (Y axis) against a decision makers hypothetical willingness to pay for every malaria episode averted (X axis). A willingness to pay at least US$67 to avert a malaria episode appears the minimum investment needed to improve on the placebo (i.e. the current practice of routine treatment and no IPTc). A willingness to pay of US$77 per episode averted shows AS+AQ monthly as the most cost effective strategy. The cost effectiveness plane presented in Figure 2 shows AS+AQ monthly as the dominant strategy; able to avert more malaria cases for less cost than both IPTc SP bimonthly and AS+AQ bimonthly.

### Scaling Up IPTc

The costs of delivering IPTc presented here are based on a well funded, small scale research study. In an attempt to better understand the costs of operationalising this intervention on a district wide scale, a model was develop to scale up costs (both fixed and variable) and explore potential savings from economies of scale. Costs reflect the amount required by the health care system to implement IPTc in the whole district with an estimated total of 33,000 children aged less than five years. To try and calculate a realistic level of IPTc coverage the total number of children in the district under five is multiplied by 74% to reflect the coverage rate reported for Ivermectin delivery (the only other recorded community health volunteer led intervention distributing medication, albeit to non pregnant women and persons aged five years and above [23]). This figure was then multiplied by the adherence rate in each of the trial IPTc drug regimen arms: 85% for SP bimonthly, 89% for AS & AQ monthly, and 88% for AS & AQ bimonthly . The assumptions for the scale up are provided in Table 6 and the unit costs of delivering the intervention district wide are reported in Table 7. The cost per child enrolled fell considerably when modelled to district level as compared to under trial conditions. For SP, the unit costs fell from US$8.19 to US$ 1.86 (a fall of 77%), for AS & AQ monthly the costs fell from US$14.79 to US$4.33 (a fall of 71%) and for AS& AQ bimonthly costs fell from US$10.67 to US$2.69 (a fall of 75%). Based on these unit costs and intervention costs, this translates to US$28.23 (19.97, 42.11, CI 95%) per malaria case averted using SP, US$22.31 (20.30, 24.54, CI 95%) using AS & AQ monthly and US$59.61 (36.40, 113.57) using AS & AQ bimonthly. As the population increases by more than forty times, the costs fall on average four times. This is due to certain fixed costs such as incentives to CBV and facility based staff remaining constant regardless of the number of children who receive IPTc. Semi fixed costs such as training, drug delivery and supervision benefited from economies of scale. In this modelling exercise the efficacy of IPTc has been assumed to be the same as that observed in the clinical trial although it cannot be guaranteed that this would be the case.

**Table 6:** Resource use when scaling up from Pilot Study to District Wide delivery.

Recurrent Costs of delivering and supervising IPTc	Pilot Study	Scale up to District Wide
Number of children receiving IPTc	2451	33,000
Cost of drugs IPTc per child		
AS+AQ monthly (1tab/day during 3 days/month for 6 months)	1.90	1.90
AS+AQ bimonthly (1 tab/day during 3days every 2 month for 6 months)	0.95	0.95
SP (one tab/day/bimonthly for 6 months)	0.06	0.06
**Personnel**		
*Drug Administration CBVs*		
Total Number of Communities	30	150
Total Number of CBVs involved in IPTc	48	150
Number of days each CBV involved in IPTc per month	3	3
Daily Incentive	US$ 3.77	US$ 3.77
*District Medical Health Team Supervision & Delivery*		
*District Director of Health Services proportion of DHS salary as ‘buy in’*	same regardless of no. of children enrolled –5% of their total salary	
Senior Nurse employed full time to coordinate all IPTc related activities district wide	-	1
Number of people involved in IPTc per month for supervision and delivery	2	10
Number of days per month each team member spends supervising IPTc	3	3
Number of days per month each team member spends delivering drugs for IPTc	2	2
Incentive over and above the salary of those involved in supervising and delivering IPTc per month	US$13.08	US$13.08
*Driver (to deliver/distribute drugs and supplies)*		
Number of Drivers involved in IPTc	2	5
Total Number of days involved	5	5
Incentive over and above the salary of those involved in driving IPTc per month	None	None
**Transport**		
Days of Vehicle use for drug delivery and supervision per month	24	4
**Training**		
Number of senior nurses/doctors involved in giving IPTc training	2	6
Time CBVs spent receiving training	2	1
Number CBVs involved in receiving training	48	150
Total Per Diem for each CBVs attending the training	US$ 12.01	US$ 6.01
Time DTHM staff involved in receiving sensitisation and training	4	2
Number DHTM staff involved in receiving sensitisation and training	58	58
Total Per Diem for each DHTM staff attending training	US$ 12.01	US$ 12.01

**Table 7:** Unit Costs of Delivering IPTc after scaling up district wide (under five population 33,000) (US$ 2008).

	SP Bimonthly		AQ & AS Monthly		AQ & AS Bimonthly	
	Economic Unit Cost	Cost Profile	Economic Unit Cost	Cost Profile	Economic Unit Cost	Cost Profile
Cost of IPTc Drugs	0.13	7%	2.04	47%	1.02	38%
Drug Administration (CBVs)	0.25	13%	0.47	11%	0.24	9%
Drug Delivery						
* Personnel*	0.16	9%	0.32	7%	0.16	6%
* Transport*	0.03	2%	0.06	1%	0.03	1%
Supervision						
* Personnel*	0.43	23%	0.58	14%	0.41	15%
* Transport*	0.01	0%	0.05	1%	0.02	1%
Training						
*CBVs*	0.19	10%	0.19	4%	0.19	7%
* DHMT and surveillance staff*	0.54	29%	0.51	12%	0.52	19%
Supplies	0.12	6%	0.11	3%	0.11	4%
**Total Cost:**	**1.86**	**100%**	**4.33**	**100%**	**2.69**	**100%**

## Discussion

At between US$8.19 and US$14.79 the annual cost of delivering at least the first one dose of each course of IPTc under trial conditions is higher than that of other interventions designed to protect children against malaria. However, when the unit costs are scaled up to a district wide level, costs of delivery fall to between US$1.86 and US$4.33 per child; these costs are within the range of the costs associated with delivering existing interventions. For example, the costs per year of protection in US$ 2008 associated with insecticide treated nets (ITNs) are reported to be US$1.46–4.00 [24], US$3.62–6.06 for indoor residual spraying (IRS)[25], US$0.75 for intermittent treatment of malaria (IPT) in infants using SP [26], US$2.02 for IPT in school children [27] and US$2.70 when delivering 2 doses of IPT to pregnant woman (using SP) via community care and US$2.39 via health centres[28]. Comparisons of ICERs across studies should be interpreted with caution due to methodological differences (e.g. some take account of resource savings and some do not, some take a societal perspective while others take a provider perspective), cultural and epidemiological profiles may differ. With this in mind, based on intervention costs alone, the cost of averting an episode of malaria in Hohoe with IPTc is high (US$67.77) compared to other malaria interventions which report the cost per malaria episode averted among under fives (in US$ 2008) of between US$3.71 for ITNs [29] and US$24.00 to US$26.58 for IRS [30].

Twenty years ago in The Gambia, mass chemoprophylaxis with Maloprim administered over several years by primary health care workers, with the support of village volunteers, to children aged 3–59 months reduced both malaria mortality and morbidity. When inflated to US$ 2008, the cost per child protected per season was US$ 4.75; the cost per childhood death averted was $239[31].

The high IPTc intervention costs in Hohoe are in part due to the small scale and vertical nature of the study. If IPTc were to be implemented routinely there is a greater likelihood of increased shared costs as costs associated with IPTc specific supervision and drug delivery could be expanded to include other non IPTc activities that would help establish a CBV network [32]. The combination of SP & AQ used in several other studies of IPTc is likely to be more cost effective because of the lower costs of SP than AS and similar or higher levels of protective efficacy [12]. This study was not powered to detect an impact on hospital admissions or mortality. However, subsequent larger studies of IPTc with SP & AQ conducted in Burkina Faso and Mali have shown a substantial reduction in hospital admissions with malaria in children who received IPTc [33] suggesting increased cost effectiveness.

As reported previously [14] the overall incidence of malaria was lower during the rainy season following the intervention than during the intervention period. The reason for this reduction in the incidence of malaria during the post intervention period is not clear. Kweku and colleagues speculate that the high coverage of IPTc, effective treatment and an increase in the use of ITNs in the study area may have led to a reduction in the transmission of malaria. The study did not suggest that there is a significant risk of rebound of malaria if IPTc is given for just one year, however, it will be important to determine whether this is also the case if IPTc is given for a longer period.

This economic evaluation shows the importance of including societal cost savings when deciding on the value of an intervention. Based on intervention costs alone IPTc may appear costly, however, once the savings to the health system and to households are included IPTc appears more favourable. The cost savings would have been more pronounced had there been more of an impact on severe episodes as has been observed in larger studies conducted in Burkina Faso and Mali[33].

Central to the success of IPTc is the sustainability of the community health volunteer network. This study has shown that this network can be sustained for a six month study but challenges may appear if this network of people is to work effectively year on year and on a larger scale. The challenges of scaling up a community – based health planning and services (CHPS) initiative in Ghana from an experimental project to a national program have been discussed elsewhere, and if IPTc is to be launched on a larger scale in Ghana, those responsible for its implementation could learn from some of the constraints to scaling up identified in the CHPS study [34], [35]. Constraints such as the time lag between the onset of planning and the actual launch of services, as well as discrepancies between the knowledge about the intervention held by the different stakeholders and their perceived roles and responsibilities, posed challenges. The need for additional resources at the primary health care level in Ghana posed problems, as staff, materials and supplies were already severely constrained. Finally a technical gap was identified; this referred to the understandable reluctance of district medical teams to launch programmes that they feared would require technical skills (such as logistical system and management information systems) not currently available. Even amid these challenges the CHPS Initiative reported take up over a 2 year period in 104 out of 110 districts in Ghana [34].

Increasing attention is being given to the role of community health workers and community health volunteers in public health programmes [36], [37]. In Ghana, a national community health volunteer network does not exist. Other programmes that rely on the services of volunteers tend to including health workers, teachers, students and community members who are willing to participate. CBVs have been studied recently in Ghana in the context of home based management of malaria (HMM) [38]–[40]. In HMM, as for IPTc, it is the responsibility of community based volunteer to dispense artemisinin based antimalarial drugs among their community and it may be feasible for CBVs to take on both roles – providing treatment whenever this is needed and giving IPTc during the period of maximum risk of malaria. The feasibility and acceptability of the CBV based HMM strategy in Ghana seems positive [41] and adherence to the multi dose drug regimen appears high [41], [42] which is encouraging for IPTc which also relies on a clear understanding by both the CBV and the caretaker of the child, as to the correct dosage of antimalarials. There is a concern, however, that confusion might arise if a CBV is seen as a dispenser of potentially the same drug for both malaria treatment and prevention. For this, and other reasons, it may be best to use different drug combinations for IPTc and for first line treatment.

The recognition that the CBV needs to be compensated was central to the success of this study and this has been reported as an important factor in other successful community health interventions [41]. In this study, a payment of approximately $10 was given to the CBV per month for a period of 6 months. This sum may be too high as it is more generous than the watches, raincoats, torches, t-shirts and $8 given every quarter to CBVs involved in the HMM. This could have implications on the equilibrium market price of CBV services.

The literature on the costs of scaling up health interventions is scarce and difficult to compare across studies [43]. When modeling the costs of scaling up IPTc, care was taken to follow some of the guiding principles set out by Johns and Tan Torres (2005): (1) fixed and variable costs were identified and scaled up to reflect economies and diseconomies of scale, (2) the availably of human resources, in this case community health workers and trained health professionals needed to oversee delivery, was assessed and thought to be realistic, (3) administrative costs were given specific attention and not assumed to remain constant. The scale up of costs did, however, fail to identify the impact of an urban delivery network as the community health workers were considered rural, in addition intra-country variation in costs and cost effectiveness were not considered[44].

This study is one of the few conducted in Ghana that has investigated the costs of treating malaria from both the provider and household perspective and the only one to use primary cost data to calculate the costs of treating severe malaria and anemia. The provider costs of malaria treatment in this study are comparable to the costs presented for WHO Choice Ghanaian inpatient visits and slightly lower for outpatient visits (having taken into account that drug and diagnostic costs have to be added to CHOICE country specific bed day cost estimates to reflect total provider treatment costs) [45]. For household expenditure on malaria treatment (direct and indirect) the results of this study appear comparable to the direct clinical/mild malaria episodes found in other estimates for Ghana [3], [46] but far higher when considering the severe and indirect costs of treatment. It is important to note the high indirect costs to households associated with inpatient care; 23–25% of total inpatient costs. A child's malaria episode has indirect costs associated with a reduction in a caretaker's paid and unpaid production. There is no consensus on how micro-economic tools help best reflect these costs [47]–[50]. The main professions in the study area were subsistence farming, gardening or domestic activities; to assume no costs were associated with these activities would underestimate the economic impact of childhood malaria on households. Therefore, formally-paid wages were used as proxies for unpaid work. All indirect costs were considered to contribute to income whether the products were eventually sold or consumed within the household, as has been done elsewhere [51], [52]. The costs of treatment in this study are most pronounced in the treatment of severe malaria and severe anaemia. This may in part be due to the small sample of households available for interview in this study. The authors of this study are not aware of any costing study using primary data that has looked at the costs of treating this group (severe malaria and anaemia) so comparisons are hard to draw. The costs of treating malaria and anaemia were modelled separately in Tanzania. Provider costs of managing cases of severe anemia and clinical malaria in infants were US$ (2008) $22.45 and $20.11 respectively. Household costs per episode of severe anemia and clinical malaria were each US$6.20 [53]. These costs were higher than the costs found in Hohoe for clinical malaria and significantly lower for the treatment costs of severe malaria.

IPTc has proved to be a safe and effective approach to reduction of the burden of malaria in Hohoe, an area of Ghana with a prolonged, intense malaria transmission season[14]. In this paper we also demonstrate how cost-effective the intervention is using three different drug regimens and the possibilities for reducing costs further if the intervention was to be scaled up to the district level. Supervision, training and remuneration of CBVs and ensuring IPTc drug delivery are identified as the main cost components and key determinants to the success of the delivery strategy.
